# Vitamin B12 deficiency in children in a high-income country: clinical features and outcomes from a 10-year retrospective study

**DOI:** 10.3389/fped.2026.1951202

**Published:** 2026-10-02

**Authors:** Riccardo Pagani, Marco Salvi, Davide Colombo, Martina Paredi, Fabiola Guerra, Adriana Cristina Balduzzi, Giulia Maria Ferrari, Paola Corti

**Affiliations:** 1 School of Medicine and Surgery, University of Milano Bicocca, Monza, Italy; 2 Department of Pediatrics, Fondazione IRCCS San Gerardo dei Tintori, Monza, Italy

**Keywords:** megaloblastic anemia, neurologic manifestations, pancytopenia, pediatric hematology, vitamin B12 deficiency

## Abstract

**Background:**

Vitamin B12 deficiency in children can cause severe hematological and neurological complications, yet epidemiological data from high-income countries remain limited. This study aimed to characterize the clinical, hematological, and etiological features of vitamin B12 deficiency in children referred to a pediatric hematology center in a high-income country.

**Methods:**

A retrospective single-center study was conducted at a pediatric hematology clinic in Northern Italy, analyzing data collected over a 10-year period. Children with serum vitamin B12 levels ≤250 pg/mL were included. Patients were stratified by total vitamin B12 into very low (≤100 pg/mL), low (101–200 pg/mL), and marginal (201–250 pg/mL) B12 levels. Clinical, laboratory, and etiological data were analyzed.

**Results:**

Of 461 eligible patients, 8.6% had serum B12 levels ≤250 pg/mL. The median age was 12 years, with a bimodal distribution peaking in infants/toddlers (35%) and adolescents (53%). The main referral reasons were anemia (52.5%), hemoglobinopathy traits (15%), isolated macrocytosis (10%), pancytopenia (12.5%), and CNS involvement (17.5%). Patients with B12 levels ≤100 pg/mL had significantly lower hemoglobin concentrations and higher biochemical markers of ineffective erythropoiesis compared to other groups.

**Conclusions:**

Vitamin B12 deficiency should be systematically considered in the differential diagnosis of unexplained cytopenias and neurological manifestations in pediatric patients, particularly in specialized hematology settings. In our cohort, serum B12 concentrations ≤100 pg/mL were associated with more pronounced hematological abnormalities and frequently observed among patients with neurological involvement; however, this finding does not establish a validated pediatric severity threshold. Early recognition and prompt treatment are essential to prevent potentially irreversible complications.

## 1 Introduction

Vitamin B12 (cobalamin) is an essential water-soluble vitamin involved in cellular replication and mitochondrial metabolism. It is critical during childhood, when rapid growth and central nervous system (CNS) development require adequate levels for normal myelination (1).

Vitamin B12 is mainly obtained from animal-derived foods and stored in the liver. Hepatic stores can delay the clinical onset of deficiency, an effect more pronounced in older children than in infants, whose limited stores are rapidly depleted (2, 3). Deficiency affects multiple organ systems, causing megaloblastic anemia, pancytopenia in severe cases, and neurological manifestations ranging from hypotonia and developmental regression in infants to cognitive and sensory symptoms in older children (1, 4–6).

Vitamin B12 deficiency is more prevalent in low- and middle-income countries (LMICs) (7–11), whereas data from high-income countries (HICs) remain limited and mainly derived from selected high-risk populations, including breastfed infants of deficient mothers and children with restrictive diets or malabsorption. This retrospective cross-sectional study evaluated the frequency, clinical presentation, and hematological features of low vitamin B12 status in children and adolescents referred to a specialized pediatric hematology clinic in Northern Italy over a 10-year period, and aimed to identify clinical contexts in which deficiency should be suspected.

## 2 Methods

### 2.1 Study population

Patients aged 30 days to 18 years evaluated at the Pediatric Hematology Clinic of Fondazione IRCCS San Gerardo dei Tintori (Monza, Italy) between January 2015 and January 2025 who underwent serum vitamin B12 measurement were included. Testing was performed in patients with suggestive hematological alterations, a clinical or dietary history indicative of nutritional deficiency, or suspected malabsorption. Patients with known chronic hematological disorders were excluded. For subgroup analyses, patients were stratified into infants and toddlers (0–2 years), children (3–10 years), and adolescents (>10 years).

### 2.2 Definition of vitamin B12 status

Vitamin B12 status was classified by total serum concentration into very low (≤100 pg/mL), low (101–200 pg/mL), and marginal (201–250 pg/mL) vitamin B12 levels. The 200 pg/mL (≈148 pmol/L) was adopted as the primary cutoff for low vitamin B12 status, in keeping with commonly used biochemical criteria, while ≤100 pg/mL (≤73.8 pmol/L) was used as an operational threshold to denote very low B12 levels, as extremely low concentrations have been associated with overt clinical and hematological manifestations (1). The 201–250 pg/mL band was used to capture potentially subclinical status, given that no standardized borderline category exists, and marginal vitamin B12 levels have been proposed in the range of 148–221 pmol/L (≈200–300 pg/mL) (11, 12).

### 2.3 Data collection

Clinical, demographic, and laboratory data were retrospectively collected from medical records. Variables included age, sex, ethnicity, dietary habits, relevant medical history, presenting symptoms, and neurodevelopmental assessments. Where neurological involvement was suspected, brain magnetic resonance imaging (MRI) was performed per routine practice, and follow-up neurological data were collected. Laboratory parameters included hemoglobin (Hb), red cell distribution width (RDW), mean corpuscular volume (MCV), serum folate, ferritin, transferrin saturation (TSAT), lactate dehydrogenase (LDH), and total bilirubin, recorded at the time of vitamin B12 measurement or the closest point before supplementation. Patients with hemoglobinopathy carrier states were retained, but their MCV and RDW values were omitted because of confounding effects on red cell indices.

### 2.4 Statistical analysis

Continuous variables are reported as mean ± SD, or as median (interquartile range, IQR) for non-normally distributed variables, and categorical variables as frequencies and percentages. Normality was assessed by Shapiro–Wilk test. Comparisons across categories used one-way ANOVA or Kruskal–Wallis test with Tukey's or Dunn's *post-hoc* tests, as appropriate. All tests were two-sided, with *p* < 0.05 considered significant. Analyses and graphs were produced with GraphPad Prism 10.2.3 (GraphPad Software, San Diego, CA, USA).

## 3 Results

Among 642 patients evaluated, 181 were excluded for a pre-existing chronic hematological disorder, leaving 461 eligible. Of these, 40 (8.6%; 24 males, 16 females) had serum vitamin B12 ≤ 250 pg/mL and formed the study cohort (Figure 1A). Median age at diagnosis was 12 years (IQR 2–16), with a bimodal distribution peaking in infants/toddlers (35%) and adolescents (53%) (Figure 1B). Most patients were of European origin (70%), followed by African (10%), Asian (10%), and South American (2.5%); ethnicity was undocumented in 7.5%. By category, 8 patients had very low (≤100 pg/mL, 20%), 20 low (101–200 pg/mL, 50%), and 12 marginal (201–250 pg/mL, 30%) vitamin B12 levels.

**Figure 1:**
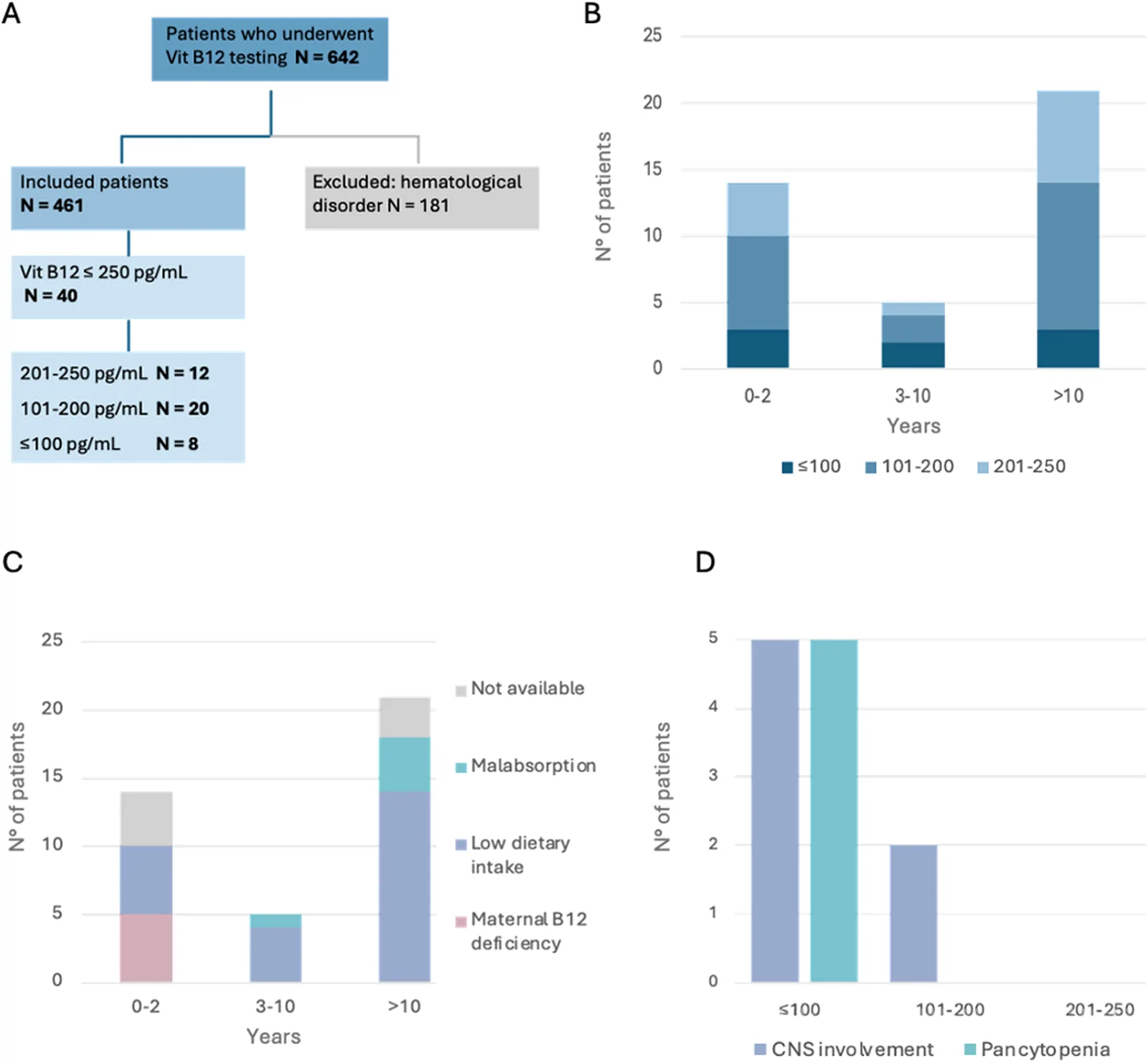
Flow diagram of cohort selection **(A)**; prevalence of vitamin B12 subgroups, defined according to serum vitamin B12 levels (pg/mL), by age group **(B)**; etiology of vitamin B12 deficiency by age group, presumed on the basis of clinical and dietary history **(C)**; prevalence of CNS involvement and pancytopenia according to vitamin B12 deficiency subgroup, defined by serum vitamin B12 levels (pg/mL) **(D)**

Underlying causes differed by age group (Figure 1C). In infants and toddlers, the likely predominant causes were maternal deficiency during breastfeeding and inadequate dietary intake; in children aged 3–10 years, low intake was the leading cause, with one case of malabsorption; in adolescents, restrictive diets predominated, followed by malabsorption, including three cases of anti-parietal cell antibody (APCA)-positive autoimmune atrophic gastritis. No inherited disorders of B12 metabolism were identified.

The main referral reasons were anemia (52.5%), hemoglobinopathy traits (15%), and isolated macrocytosis (10%). Pancytopenia occurred in 5 patients (12.5%) and CNS involvement in 7 (17.5%), co-occurring in 3 patients (7.5%). Pancytopenia was confined to the very low group. CNS manifestations occurred predominantly in infants and toddlers, mostly in the very low group (5 of 7); of the remaining 2 patients, one had a level just above the very low threshold (109 pg/mL) and the other was measured after one week of oral supplementation, which may have partially corrected the value (Figure 1D).

Clinical and laboratory characteristics by vitamin B12 status and age group are shown in Tables 1, 2; comparisons of hematological and biochemical parameters by status are illustrated in Supplementary Figure S1.

**Table 1:** Patient characteristics and their hematological and biochemical parameters according to vitamin B12 status.

Patient characteristics	Vitamin B12 ≤100 pg/mL Pts *N* = 8	Vitamin B12 101–200 pg/mL Pts *N* = 20	Vitamin B12 201–250 pg/mL Pts *N* = 12	Total Pts *N* = 40
Demographics				
Male/Female, N/N	4/4	15/5	5/7	24/16
Age at diagnosis (yrs), median (mean ± SD)	5.5 (7.1 ± 6.4)	13 (9.4 ± 6.9)	12 (9.8 ± 6.8)	12 (9.1 ± 6.7)
Origin				
European, N (%)	*N* = 5 (62.5)	*N* = 13 (65)	*N* = 10 (83.3)	*N* = 28 (70)
African, N (%)	*N* = 2 (25)	*N* = 2 (10)	*N* = 0 (0)	*N* = 4 (10)
Asian, N (%)	*N* = 0 (0)	*N* = 3 (15)	*N* = 1 (8.3)	*N* = 4 (10)
South American, N (%)	*N* = 0 (0)	*N* = 0 (0)	*N* = 1 (8.3)	*N* = 1 (2.5)
Unknown, N (%)	*N* = 1 (12.5)	*N* = 2 (10)	*N* = 0 (0)	*N* = 3 (7.5)
Etiology of B12 deficiency^a^				
Maternal B12 deficiency, N (%)	*N* = 2 (25)	*N* = 3 (15)	*N* = 0 (0)	*N* = 5 (12.5)
Low dietary intake, N (%)	*N* = 5 (62.5)	*N* = 10 (50)	*N* = 8 (66.7)	*N* = 23 (57.5)
Malabsorption, N (%)	*N* = 1 (12.5)	*N* = 3 (15)	*N* = 1 (8.3)	*N* = 5 (12.5)
Unknown, N (%)	*N* = 0 (0)	*N* = 4 (20)	*N* = 3 (25)	*N* = 7 (17.5)
Main referral reason				
Anemia, N (%)	*N* = 0 (0)	*N* = 12 (60)	*N* = 9 (75)	*N* = 21 (52.5)
Macrocytosis, N (%)	*N* = 1 (12.5)	*N* = 3 (15)	*N* = 0 (0)	*N* = 4 (10)
Hemoglobinopathy traits, N (%)	*N* = 0 (0)	*N* = 3 (15)	*N* = 3 (25)	*N* = 6 (15)
Pancytopenia, N (%)	*N* = 5 (62.5)	*N* = 0 (0)	*N* = 0 (0)	*N* = 5 (12.5)
CNS involvement, N (%)	*N* = 5 (62.5)	*N* = 2 (10)	*N* = 0 (0)	*N* = 7 (17.5)
Hematological and biochemical characteristics				
Hb (g/dL), median (mean ± SD)	4.3 (5.8 ± 3.1)	8.6 (9.1 ± 3.1)	9.2 (9.6 ± 3.1)	8.1 (8.6 ± 3.5)
MCV (fL), median (mean ± SD)	99.1 (96.8 ± 8.8)	78.1 (77.3 ± 16.8)	66.9 (67.7 ± 13.8)	81.9 (79 ± 17.7)
RDW (%), median (mean ± SD)	22.4 (21.7 ± 5.2)	17.7 (18.4 ± 4.5)	13 (13.9 ± 2.1)	17.5 (18.5 ± 5.1)
LDH (U/L), median (mean ± SD)	2,931 (3,949 ± 2,618)	306.5 (367.7 ± 137.8)	249 (235 ± 42.2)	436 (1,442.3 ± 2,254.9)
Bilirubin (mg/dL), median (mean ± SD)	1.4 (1.5 ± 0.6)	0.3 (0.5 ± 0.5)	0.5 (0.56 ± 0.3)	0.5 (0.8 ± 0.6)
Ferritin (ng/mL), median (mean ± SD)	163.5 (153 ± 92.5)	17 (61.1 ± 81.3)	6.9 (26.5 ± 46.9)	40.5 (59.9 ± 51.5)
TSAT (%), median (mean ± SD)	67.2 (55.5 ± 33)	12.9 (15.6 ± 12.8)	4.4 (9.1 ± 9.5)	9.5 (20.4 ± 22.9)
Folate (ng/mL), median (mean ± SD)	4.2 (9.9 ± 10.9)	4.5 (7.2 ± 6)	3.7 (4.6 ± 3.4)	4.1 (7 ± 7)

a Etiology was presumed on the basis of clinical and dietary history.

**Table 2:** Patient characteristics according to age group.

Patient characteristics	Age 0–2 yrs Pts *N* = 14	Age 3–10 yrs Pts *N* = 5	Age >10 yrs Pts *N* = 21
Etiology of B12 deficiency^a^			
Maternal B12 deficiency, N (%)	*N* = 5 (35.7)	*N* = 0 (0)	*N* = 0 (0)
Low dietary intake, N (%)	*N* = 5 (35.7)	*N* = 4 (80)	*N* = 14 (66.7)
Malabsorption, N (%)	*N* = 0 (0)	*N* = 1 (20)	*N* = 4 (19)
Unknown, N (%)	*N* = 4 (28.6)	*N* = 0 (0)	*N* = 3 (14.3)
Main referral reason			
Anemia, N (%)	*N* = 9 (64)	*N* = 2 (40)	*N* = 10 (47.6)
Macrocytosis, N (%)	*N* = 0 (0)	*N* = 1 (20)	*N* = 3 (14.3)
Hemoglobinopathy traits, N (%)	*N* = 0 (0)	*N* = 0 (0)	*N* = 6 (28.6)
Pancytopenia, N (%)	*N* = 1 (7)	*N* = 2 (40)	*N* = 2 (9.5)
CNS involvement, N (%)	*N* = 5 (35.7)	*N* = 2 (40)	*N* = 0 (0)

a Etiology was presumed on the basis of clinical and dietary history.

Treatment was initiated in all patients with very low vitamin B12 levels, administered parenterally in 7 of 8 (87.5%), with the route determined by clinical presentation and treating-physician assessment. In the low group, supplementation was prescribed in 19 of 20 patients (95%), parenteral in 8 of 19 (42%). In the marginal group, supplementation was prescribed in 3 of 12 patients (25%). Among the three adolescents with APCA-positive autoimmune atrophic gastritis, one received parenteral cobalamin from diagnosis, while the other two were switched to parenteral treatment after an inadequate response to oral supplementation.

## 4 Discussion

### 4.1 Epidemiology and etiology

In this retrospective study performed in Northern Italy, 8.6% of evaluated patients had vitamin B12 levels ≤250 pg/mL. This frequency cannot be extrapolated to the general pediatric population because testing was based on clinical suspicion, but it provides relevant data from a HIC setting where pediatric deficiency remains insufficiently characterized. The bimodal age distribution, peaking in infants/toddlers and adolescents, is consistent with previous reports. In infancy, risk was likely related mainly to maternal deficiency during breastfeeding and inadequate complementary feeding; in older children, restrictive diets and malabsorption contributed substantially. Three adolescent cases of autoimmune atrophic gastritis indicate that pernicious anemia, traditionally considered an adult-onset disorder, can present in adolescence and should therefore be considered in the differential diagnosis of vitamin B12 deficiency. No inherited disorders were observed, likely reflecting their rarity and the referral population.

### 4.2 Hematological and neurological manifestations

Pancytopenia occurred in 5 patients (12.5%), exclusively in the very low group, likely reflecting referral bias inherent to a specialized pediatric hematology setting. Incidence rates of pancytopenia in pediatric vitamin B12 deficiency are not well established, and the condition is described mainly through case reports. Demirci et al. (13), in 10,268 children, found B12 deficiency predominantly associated with mild hemogram abnormalities and no cases of pancytopenia. Its occurrence across age groups indicates that severe hematological manifestations may develop when deficiency is sufficiently profound. Vitamin B12 deficiency should therefore always be considered in unexplained cytopenias, especially with macrocytosis or evidence of ineffective erythropoiesis.

CNS involvement occurred in 7 patients (17.5%), predominantly in infants and toddlers (71.4% of CNS cases) and strongly associated with very low vitamin B12 levels, consistent with the vulnerability of the developing nervous system. Manifestations included psychomotor developmental delay, lethargy, persistent vomiting, and hypotonia; brain MRI ranged from delayed myelination to cerebral atrophy with enlarged cerebrospinal fluid spaces. This age group poses a diagnostic challenge, as signs such as failure to thrive or motor regression may emerge only after deficiency is advanced, reinforcing the need for a low threshold of clinical suspicion in infants and toddlers.

All patients showed rapid clinical improvement after supplementation, and short-term follow-up confirmed favorable neurodevelopmental outcomes. Nevertheless, the long-term prognosis of infantile deficiency remains concerning. Kumar et al. (14) prospectively followed 141 infants and found that, despite improvement after supplementation, 67.5% had developmental retardation at follow-up, indicating that duration of deficiency before treatment is possibly the most important prognostic factor. Newborn screening programs have indeed shown potential to prevent symptomatic infantile deficiency through early detection (15), underscoring the value of timely treatment in preventing irreversible neurological sequelae.

### 4.3 Laboratory findings

Differences across categories reflect the pathophysiology of deficiency. Lower Hb and increased MCV and RDW in the very low group reflect impaired DNA synthesis in proliferating erythroid precursors, producing classical megaloblastic changes. Elevated LDH and bilirubin are consistent with ineffective erythropoiesis and intramedullary hemolysis, while increased ferritin and TSAT may reflect iron release from intramedullary apoptosis of erythroid cells with reduced hemoglobin incorporation. The trend toward higher folate in the very low B12 group may reflect the “methyl-folate trap” mechanism (16). A clear separation was seen between the very low group (≤100 pg/mL) and higher categories, consistent with a possible severity gradient at this concentration; fewer differences emerged between the low and marginal groups. Given the small number of patients in these strata, this observation should be interpreted as hypothesis-generating rather than as evidence of equivalence, as the study was underpowered to detect modest between-group differences.

### 4.4 Limitations

Serum vitamin B12 cut-offs in children are not standardized, and most thresholds derive from adult studies. Total serum B12 is age-dependent, with higher concentrations in infants and young children, and age-specific pediatric reference intervals have been proposed (17, 18). However, these intervals were derived from different populations, age partitions, and are not fully concordant. Fixed thresholds were therefore retained as predefined operational categories to ensure consistent cohort definition and comparability with existing literature, while their limitations are acknowledged. Inclusion was limited to tested patients, and testing based on clinical suspicion may have overestimated prevalence. The retrospective design relied on records that may be incomplete: quantitative B12 dietary intake could not be estimated, and although maternal deficiency was documented, specific maternal B12 values could not be retrieved. Functional biomarkers (methylmalonic acid, homocysteine, holotranscobalamin) were not routinely assessed, potentially reducing diagnostic accuracy. Finally, the small sample size limited the power of subgroup analyses.

## 5 Conclusion

Vitamin B12 deficiency should be suspected in children with unexplained cytopenias or neurological and neuropsychiatric involvement, particularly in the pediatric hematology setting. Key scenarios include exclusively breastfed infants with developmental delay or feeding difficulties and adolescents with unexplained anemia or macrocytosis, notably with restrictive diets. Although often regarded as a problem of low-resource or immigrant populations, its occurrence in this predominantly European cohort shows that the burden in high-income countries is not negligible. In our cohort, serum vitamin B12 concentrations ≤100 pg/mL were associated with more pronounced hematological abnormalities and were frequently observed among patients with neurological involvement; however, this finding should be considered exploratory and does not establish a validated pediatric severity threshold. Rather than relying on any single threshold, serum vitamin B12 should be interpreted alongside age, clinical presentation, dietary history, and hematological and neurological findings, ideally complemented by functional biomarkers, so that early recognition and timely treatment can prevent potentially irreversible sequelae.

## Data Availability

The data analyzed in this study is subject to the following licenses/restrictions: Access to the dataset is restricted due to patient confidentiality, ethical requirements, and data protection regulations. The dataset contains individual-level clinical and laboratory information from pediatric patients and cannot be publicly released. Access to these datasets is not available due to ethical and privacy restrictions regarding patient-level clinical information. Requests to access the datasets should be directed to the corresponding author.

## Supplementary material

Supplementary Figure S1 Comparison of hematological and biochemical characteristics of patients according to vitamin B12 status. Data are expressed as mean ± standard deviation. Statistical significance is indicated as follows: **p* < 0.05, ***p* < 0.01, ****p* < 0.001.
